# In memoriam: Dr Brighid Malone

**DOI:** 10.4102/phcfm.v18i1.5815

**Published:** 2026-10-23

**Authors:** Billy M. Tsima, Claire L. Brockbank

**Affiliations:** 1 Department of Family Medicine and Primary Health Care, School of Medicine, University of Botswana, Gaborone, Botswana

Dr Brighid Malone, a distinguished Family Physician, mentor, dedicated clinician, founding member of the Botswana Association of Family Physicians and member of Botswana’s medical community passed away on 19 August 2026.

Born in Ireland on 26 December 1960, she completed her medical training at Trinity College Dublin and obtained the Bachelor of Medicine, Surgery and Obstetrics (MB BCh BAO) degree in 1984. She subsequently pursued postgraduate training in general practice in Ireland and Scotland between 1987 and 1989, ultimately becoming a Member of the Royal College of General Practitioners in the United Kingdom.

Dr Malone was a permanent resident of Botswana who devoted over 30 years of her professional life to the care of others. She moved to Botswana from Zimbabwe in 1992 after serving as a Medical Officer at Chinhoyi Provincial Hospital for 2 years. She served as a Senior Medical Officer in Obstetrics and Gynaecology at Gaborone Private Hospital (1992–1994) and subsequently practised as a Family Physician at Independence Surgery, a private clinic in Gaborone and later joined Bokamoso Private Hospital.

She dedicated many years of service to the Botswana Health Professions Council, taking on multiple assignments and contributing to the development of professional standards, regulatory processes and the growth of medical practice in Botswana.

Dr Malone’s contribution to Family Medicine in Botswana is noteworthy. As a pioneer of the discipline in the country, she recognised the need to ‘grow own timber’ and thus dedicated countless hours to mentoring aspiring Batswana Family Physicians. Many of whom have gone on to be changemakers, driving improvements in patient-centred care in Botswana. Dr Malone also served as an external examiner for the University of Botswana Family Medicine programme.

Service to others was important to Dr Malone, often volunteering her time as a physician to serve vulnerable communities such as children and adults living with human immunodeficiency virus (HIV) at The Maipelo Trust. As a member of the Botswana HIV Clinicians Society, her contribution to the country’s laudable achievements in HIV care is remarkable. As a long-standing member of the Diabetes Association of Botswana, she constantly advocated for quality diabetes care. She utilised her own experience as a cancer survivor to bring wisdom and advocacy to her role at Journey of Hope Botswana. Thus, showing true humility and empathy, which also reflected in her clinical practice and her work with colleagues.

Dr Malone’s passing represents a significant loss to Botswana’s medical community, yet her legacy lives on in the patients she cared for, the doctors she mentored, the institutions and communities she benevolently served.

